# Head and Neck Cancer in the Elderly: A Retrospective Study over 10 Years (1999 - 2008)

**DOI:** 10.1186/1758-3284-2-25

**Published:** 2010-10-05

**Authors:** Astrid L Kruse, Marius Bredell, Heinz T Luebbers, Klaus W Grätz

**Affiliations:** 1 Department of Craniomaxillofacial and Oral Surgery, University of Zurich, Switzerland

## Abstract

**Introduction:**

Treatment of elderly patients is in many ways different from that for younger ones. The aim of the present study was to identify the particular characteristics and needs of elderly patients suffering from head and neck cancer. From these patterns, considerations for this special group can be deduced.

**Patients and Material:**

The subjects for this study consisted of 376 patients suffering from head and neck cancer that were treated between 1999 and 2008, 99 (26.3.%) of whom were older than 70 years and were evaluated retrospectively concerning smoking/alcohol abuse, ASA status, kind of malignant neoplasm, localization and treatment.

**Results:**

The male-female ratio was 53:46, and mean age, 79 years (71 - 98). Out of 95 patients with a squamous cell carcinoma, 4 patients had a verrucous form. Out of 99 patients, 26 had a maxillary carcinoma and 12 patients had experienced previous non-head-and-neck cancer. An ASA score of 2 or 3 was found in 86 of the patients.

**Conclusion:**

The group of patients with head and neck cancer who were older than 70 years was characterized by a higher portion of female patients, a higher number of maxillary carcinomas, and a higher prevalence of previous second cancer.

Making decisions in cancer therapy for elderly patients is challenging. Patients suffering from operable head and neck cancer should be treated with curative intent and with regard to quality of life if a careful assessment of comorbidities is performed preoperatively.

## Introduction

The percentage of elderly people with head and neck cancer is rising due to an overall increase in life expectancy. Age has been shown to be an independent predictor of perioperative outcome, morbidity, and mortality risk. The main risk factors for head and neck cancer can be smoking and alcohol consumption, and these factors are also often associated with cardiovascular and pulmonary comorbidities, leading to a challenge concerning treatment decisions in this patient group.

An aging heart has less efficient cardiac output under the stress of surgery and anaesthesia, leading to lower renal blood flow and possibly causing a higher sensibility for greater water and electrolyte imbalances. Furthermore, pulmonary function is compromised with increased age due to smaller vital capacities and poorer gas exchange because of deterioration of the lung parenchyma (Table 1).

**Table 1:** Age-related changes according to Priebe and Lakatta [5,6].

	Influence	Age-related change
**Organ function**	Respiratory	- Increased chest wall rigidity
		- Decreased functional alveolar surface area, decreased gas exchange
		- Decreased respiratory muscle strength and endurance
	Hepatic	- Decreased activity of hepatic cholinesterase
		- Decrease in microsomal demethylation pathway
	Renal	- Reduced glomerular filtration rate
		- Decreased renal blood flow
		- Reduction in total body water
	Miscellaneous	- Reduced skeletal mass
		- Reduced cortisol secretion
	Cardiac	- Increased myocardial stiffness
		- Increased aortic impedance
		- Increased left atrium size
	Vascular	- Increased vascular stiffness
		- Decreased β-adrenoceptor responsiveness
**Drug Disposition**	Drug distribution	- Reduced total body water
		- Reduced body mass
		- Reduced serum albumin
	Renal excretion	- Reduced glomerular filtration rate
		- Reduced renal blood flow
		- Reduced concentrating ability
	Hepatic metabolism	- Reduced hepatic blood flow
		- Reduced activity of microsomal oxidizing system

In the literature, no exact age seems to be associated with the word "elderly." In any case, surgical indications should not be based on age, but rather on risk assessment. Socinski et al. defined an "old patient" as one whose health status has begun to interfere with oncological decision-making guidelines [1]. Therefore, some authors recommend using the ASA score as a tool for risk assessment [2-4].

Postoperative delirium is a common complication, with the overall incidence estimated at 10% in elderly patients following major elective non-cardiac surgery [7]. It usually presents around 24 hours postoperatively, resolving in most patients within 48 hours, but episodes can last for months [8]. With delirium the course usually fluctuates and revolves over some days, up to a week, but with dementia symptoms are progressive. Hallucinations and delusions are also often absent in dementia, unlike in delirium. Concerning therapy, Dexmedetomidine is recommended for long-term sedation in the intensive care unit (ICU) as it leads to a decreased incidence of ICU delirium [9,10], while low-dose Haloperidol or Donazepil does not reduce the incidence [11-13].

In general, preoperative risk assessment is predictive for the development of postoperative morbidity [14-16]. Serletti et al. [2] regarded surgical time longer than 10 hours as a predictive factor for development of postoperative surgical complications. Further factors are large fluid shifts and significant blood loss. Another important factor in reconstructive failure seems to be the presence of peripheral vascular disease.

Concerning chemotherapy, Shayne et al. reported that in patients ≥ 70 years who were receiving full-dose chemotherapy, increasing age alone did not increase the risk of hematologic toxicity [17].

The aim of the present study is to identify the concerns and needs of elderly patients suffering from head and neck cancer. From these patterns special considerations for this group can be deduced.

## Patients and Material

Out of 376 patients treated from 1999 through 2008 that were suffering from head and neck cancer, 99 (26.3%) were older than 70 years. This group was evaluated retrospectively concerning smoking/alcohol abuse, ASA status, kind of malignant neoplasm, location of malignancy, treatment, and outcome.

## Results

### Gender distribution, smoking, alcohol

**Figure 1:**
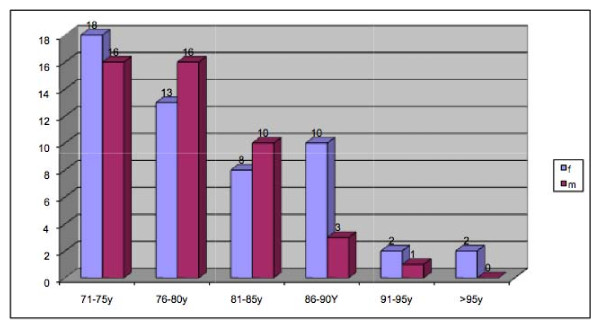
**Age distribution of all patients over 70 years**.

**Figure 2:**
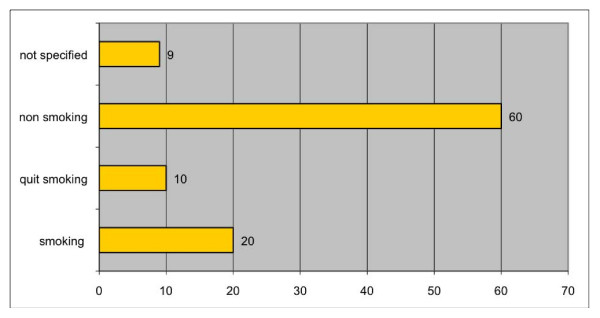
**Risk factor smoking**.

**Figure 3:**
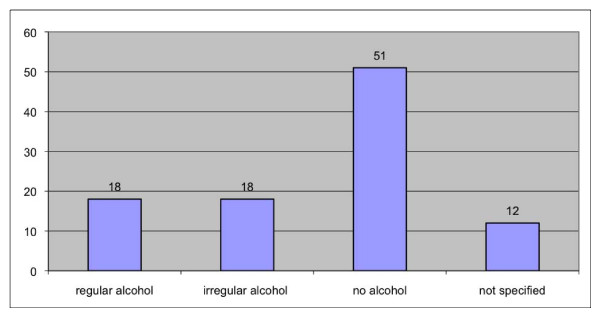
**Risk factor alcohol**.

The male-female ratio was 46:53, the mean age 79 years (71 - 98) (Figure 1). Out of 90 evaluated patients, 20 were still smoking (Figure 2), and 51 out of 77 patients said they did not consume alcohol (Figure 3).

### Kind of malignant neoplasm, location

**Figure 4:**
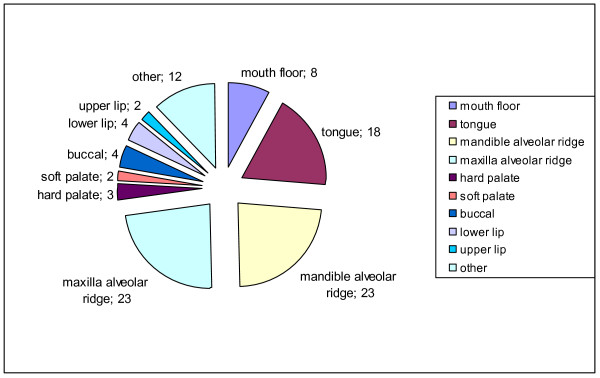
**Location of tumors**.

Out of 95 patients with a squamous cell carcinoma, 4 had a verrucous form. The male-to-female distribution of those patients with a verrucous form was 1:3; none of them developed lymph node metastases or recurrence. Five patients revealed a malignoma of the small salivary glands, 2 patients a malignant melanoma, and one a metastasis of a prostate carcinoma. The different locations of malignant neoplasms are presented in Figure 4. Almost one-third was localized in the maxilla, and the second most common location was the mandible ridge, followed by the tongue.

### ASA status, general diseases

**Figure 5:**
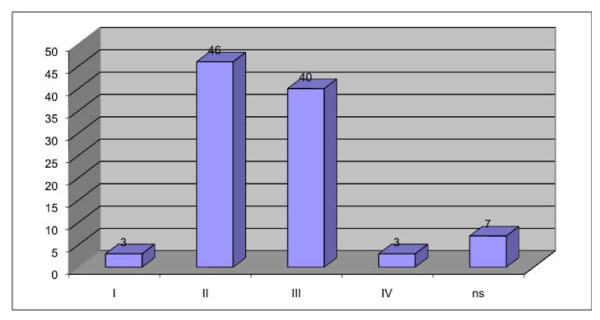
**ASA status of all patients**.

Concerning assessment of personal surgery risk, the ASA status was determined as listed in Figure 5. Out of 92 evaluated patients with an ASA score, 86 were either ASA class II or class III.

**Figure 6:**
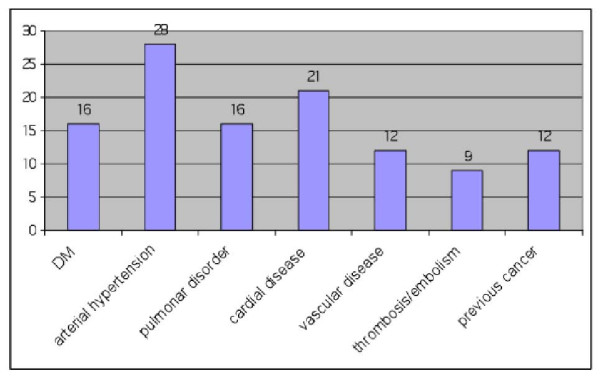
**Distribution of general diseases (DM = Diabetes mellitus)**.

Most of the patients had some type of cardiovascular disease, and in 12 patients a previous non-head-and-neck cancer was found (Figure 6).

**Figure 7:**
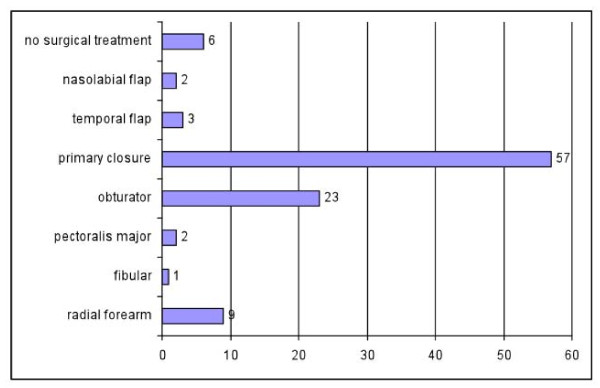
**Treatment of elderly patients**.

**Figure 8:**
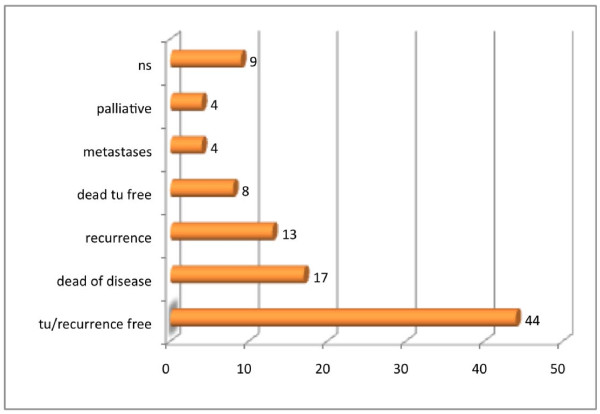
**Outcome**.

The different therapies are listed in Figure 7. The most common therapy was primary closure followed by obturator prosthesis. Concerning the outcome (Figure 8), 44 patients were recurrence free, but the median follow-up time was 25.5 months because a large portion of the elderly patients did not appear at their follow-up sessions.

## Discussion

Muir et al. estimated that as many as 24% of head and neck cancers are found in patients older than 70 years [18], and this was supported by our study. A higher female proportion, as in the present study, was also confirmed by Sarini et al. [19].

Furthermore, in the present study the rate of patients with alcohol abuse or smoking risk factors was found to be lower than that in younger patient groups. This finding follows the logic that malignant tumors occur earlier under the influence of risk factors, but are also likely to occur without them as time passes by. Malignancy may interfere with accumulation of mutations, decreased efficiency in DNA repair, and reduced immune surveillance.

Concerning the higher portion of maxillary carcinomas in elderly patients, similar results can be shown in other studies [20,21]. These findings are also important with regard to therapy planning because performing elective neck dissections for maxillary carcinoma is still controversial. It is also striking that, particularly in maxillary carcinoma, the proportion of females is higher than that of males [21].

Four patients had a verrucous form; this type of well-differentiated squamous cell carcinoma is well known in elderly patients [22].

In deciding which treatment strategy would be suitable for an individual elderly patient, a comprehensive geriatric assessment seems to be the essential step, because age by itself is an unreliable parameter for decision making. In the present study, only 3 patients had an ASA status of 1, and the predominant therapy was primary closure.

Due to changed renal function (reduced glomerular filtration rate, decreased renal blood flow) and changed hepatic metabolism (reduced hepatic blood flow and reduced activity of the microsomal oxidizing system), drug distribution is also changed in elderly patients. Moreover, increased myocardial stiffness, increased aortic impedance, increased left atrium size, and increased vascular stiffness, in addition to decreased β-adrenoceptor responsiveness, must be considered before a surgical treatment is planned for elderly patients (Table 1).

Though our population is becoming older and, therefore, the risk of developing cancer is also increasing, little research evaluating age-related toxicities has been conducted, and age-related guidelines for chemotherapy administration are often missing. The therapy decision should be based on the patient's wishes, ASA score, and combined quality of life and function, instead of being based on the patient's age and years of life expectancy.

### Perioperative mortality

In 810 patients older than 65 years who had undergone major head and neck resections under general anaesthesia, Morgan et al. reported a mortality rate as low as 3.5% [23]. In addition, Jones et al. reported no significant differences in perioperative or postoperative complications between head and neck cancer patients older than 70 years and patients younger than 66 years, although the older group had a higher frequency of morbid preoperative conditions [24]. In the present study the ASA score was applied preoperatively in order to evaluate the individual surgical risk, and none of the patients died perioperatively.

### Microvascular

Controversy still surrounds microvascular free tissue transfer. Studies dealing with free tissue in the elderly have been done for those aged 50 years [3,25], 60 years[14], 65 years[2,15], and 70 years [16]. The flap loss rate in these studies ranged from 1% out of 92 patients [25] to 16.7% out of 47 patients [14,24].

Blackwell et al. reported that microvascular reconstructions in elderly patients are reliable, but the incidence of medical complications and costs has significantly increased [25]. The success rate of free flaps seems no different from that for other age groups, but postoperative medical complications seem to be directly related to the presence of concurrent illness [3]. In the current study a microvascular reconstruction was performed in only 13 patients; in all cases it was for mandible/mouth floor or tongue reconstruction. For maxilla, the most common closure was performed by an obturator prosthesis, followed by primary closure or temporal/nasolabial flaps.

## Conclusion

Even if the concept of "elderly" has no clearly defined parameters, these patients do have a different pattern of head and neck tumors with regard to risk factors, tumor entities, and treatment options. The group of patients older than 70 years with head and neck cancer is characterized by a higher proportion of female patients, a higher number of maxillary carcinomas, and a higher prevalence of previous cancers.

Therefore the elderly patient does require an exact evaluation of his biological situation and an especially individual treatment concept.

## Conflict of interests

The authors declare that they have no competing interests.

## Authors' contributions

AK performed the retrospective study, MB and HT drafted the manuscript and KG participated in the design of the study and coordination. All authors read and approved the manuscript.
